# Indole analogs and the aryl sulfonamide AqB011 potently restrain the invasiveness of glioblastoma cells, as determined in *ex vivo* xenograft tissue slice co-cultures (XTCCs)

**DOI:** 10.1186/s12935-026-04489-8

**Published:** 2026-10-27

**Authors:** Kim K. Wagner, Andrea J. Yool, Rohan A. Davis, Katrin Becker, Heike Franke, Achim Aigner

**Affiliations:** 1 School of Pharmacy and Biomedical Science, College of Health, Adelaide University, Adelaide, 5005 Australia; 2 Rudolf-Boehm-Institute for Pharmacology and Toxicology, Medical Faculty, University of Leipzig, Härtelstrasse 16-18, Leipzig, D-04107 Germany; 3 Rudolf-Boehm-Institute for Pharmacology and Toxicology, Clinical Pharmacology, Medical Faculty, University of Leipzig, Härtelstrasse 16-18, Leipzig, D-04107 Germany; 4 Institute for Biomedicine and Glycomics, Griffith University, Nathan, 4111 QLD Australia; 5 School of Environment and Science, Griffith University, Nathan, 4111 QLD Australia; 6 NatureBank, Griffith University, Nathan, 4111 QLD Australia

**Keywords:** Indolamide, Semisynthetic indole analogs, Aquaporin, Glioblastoma, Invasion, Tissue slice co-culture models

## Abstract

**Background:**

Glioblastomas (GBMs) are highly invasive and heterogeneous in nature which strongly contributes to poor survival of patients, averaging 15 months after diagnosis. With little treatment options being available in particular for addressing GBM invasiveness, there is a high need for supplementary therapies in addition to classical cytostatics like Temozolomide (TMZ).

**Methods:**

The aim of this study was to examine promising compounds, the natural indole scaffold RAD114 and three semisynthetic analogs (RAD118, RAD119, RAD120) as well as the aryl sulfonamide AqB011, for their inhibitory potential specifically on tumor cell invasion. For this, GBM xenograft tissue slice tandem co-culture (XTCC) models were employed, closely reflecting the *in vivo* patient situation. In particular, these *ex vivo* tissue slice air-liquid interface (ALI) models also allow for distinguishing between inhibitory effects on bulk tumor growth vs tumor cell invasion into the normal brain tissue.

**Results:**

Profound anti-invasive effects of the natural indole scaffold RAD114, three semisynthetic analogs (RAD118, RAD119, RAD120), and the aryl sulfonamide AqB011 were observed in XTCC models based on G55T2 and U87-MG GBM xenografts co-cultured with mouse brain slices in ALI tandem co-culture. While effects on bulk tumor growth were rather moderate, profound inhibition or even abolishment of tumor invasion was observed, dependent on the compound, its dosage and the model. At a non-toxic concentration (10 µM), RAD118 and AqB011 showed the highest effectiveness in reducing tumor invasion. Combination effects at sub-optimal compound dosages were observed as well.

**Conclusions:**

Both compound classes - the tested semisynthetic indole analogs and the sulfonamide derivative – may be promising lead candidates for drugs useful to specifically address the issue of GBM invasiveness. They strongly restrained GBM cell invasion, alone and in combination. Rather than acting through cytotoxic effects, they specifically inhibit cell migration into native brain tissue.

**Supplementary Information:**

The online version contains supplementary material available at https://doi.org/10.1186/s12935-026-04489-8.

## Introduction

Glioblastoma multiforme (GBM) is a devastating brain tumor with limited treatment options, high relapse rates, and low survival times. The aggressive nature of GBM can be attributed to its high invasiveness and heterogeneity [1–4]. This requires the development of novel pharmacological treatment strategies and the availability of powerful models for their rigorous testing.

Indole-derived compounds are a large group of diverse, naturally occurring compounds that are used in traditional and modern medicine [5] to treat multiple indications, including neurological disorders [6, 7], cardiovascular diseases [8], fibrosis [9], and cancer [10]. For example, three vinca indole alkaloids – Vincristine, Vinblastine, and Vinorelbine – are FDA-approved for lymphomas and breast cancer [10]. However, due to their nonspecific activity, vinca alkaloids are associated with severe intestinal and neurotoxic side effects [11]. The naturally occurring indole derivative Evodiamine is a common component of the dried unripe fruit of *Evodia rutaecarpa* used in Traditional Chinese Medicine [12] and known for its wide range of pharmacological activities [13]. It has been tested on several cancer types [14–16] including GBM, in which it induced autophagy and apoptosis at 6 μM [17]. The cytotoxic indole alkaloid 3α-Acetonyltabersonine was shown to induce apoptosis in GBM cell lines and GBM stem-like cells [18]; however, its blood–brain barrier (BBB) penetration and potential toxic effects on normal cells remain to be tested. Another promising drug candidate is the indole derivative ST-11 that binds to tubulin, inducing Caspase-3-mediated apoptosis in GBM cell lines [19]. Additional indole derivatives tested in GBM cell lines are oxindole-based PI3K/AKT/mTOR inhibitors, which reduce GBM cell proliferation and induce apoptosis through cell cycle arrest [20]. These compounds have shown promising anti-cancer activities focused mainly on the induction of apoptosis and inhibition of proliferation. However, since the invasiveness of GBMs is a major challenge not addressed by current therapies, we sought to identify compounds that could restrain GBM motility.

Based on a preliminary screen, we identified several indole derivatives with promising *in vitro* activity in four GBM cell lines via mechanisms that included inhibition of aquaporin-1 (AQP1) water channels [21]. The parent compound, Indole-3-carboxylic acid (RAD114), previously isolated from marine organisms and plants [22], was chemically modified to obtain the analogs RAD118, RAD119, and RAD120 [23].

*Ex vivo* xenograft tissue slice co-culture (XTCCs) models have been shown previously to enable the monitoring and visualization of the growth and invasion patterns of different types of GBM in living brain tissue [24]. This provides an advanced platform for studies aiming at the definition of mechanisms of invasion and candidate drugs for therapeutic interference [25]. XTCCs allowed the first demonstration of differential effects of GBM drug candidates on GBM tumor bulk growth and single-cell invasion into normal brain tissue [25].

In this study, we tested the new semisynthetic indole analogs (or derivatives) RAD114, RAD118, RAD119, and RAD120, as well as the aryl sulfonamide AqB011, with a particular focus on using the XTCC models.

## Materials and methods

### Cell culture

The human glioblastoma cell line U87-MG was purchased from the American Type Culture Collection (ATCC, Manassas, VA), and the human glioblastoma cell line G55T2 was kindly provided by Dr. Katrin Lamszus. U87-MG were cultured in Dulbecco Modified Eagle Medium (DMEM; Sigma-Aldrich, Steinheim, Germany) supplemented with 10% Fetal Bovine Serum (FBS) and 2 mM L-Alanyl-L-glutamine (Biochrom, Berlin, Germany). G55T2 cells were cultured in Iscove’s Modified Dulbecco’s Medium (IMDM; Sigma-Aldrich, Steinheim, Germany) supplemented with 10% FBS and 2 mM L-Alanyl-L-glutamine. Both cell lines were grown at 37 °C in a humidified 5% CO_2_ incubator. For passaging, cells were washed briefly with Phosphate-buffered saline (PBS), rinsed with Trypsin-EDTA for 5 min at 37 °C, and resuspended in the corresponding cell culture medium with supplements. The cell number was determined with a Neubauer counting chamber.

### Pharmacological agents

The indole parent compound (RAD114) and semisynthetic analogs (RAD118, RAD119, RAD120) were prepared as described previously [21]. The bumetanide derivative AqB011 (MW 434.9; ClogP 1.80) was produced by Dr. G. Flynn (Spacefill Enterprises LLC, Oro Valley, AZ) and used to selectively block the AQP1 ion channel pore [26]. For chemical structures, see Suppl. Figure 1. The dry compounds were dissolved in Dimethyl Sulfoxide (DMSO) to obtain a 10 mM stock solution (stored at 4 °C). For assays, the test compound DMSO stocks were diluted in cell culture media to a concentration of 100 µM, the highest dose tested, and serial dilutions in media were used to prepare 10 and 30 µM doses. 1% DMSO in the same medium served as the vehicle control, matching the amount present in the highest treatment dose.

### Cell viability assay

Cell viability was determined with the WST-8 Cell Proliferation Assay Kit (Sigma-Aldrich, Darmstadt, Germany) which measures mitochondrial activity. Briefly, G55T2 and U87-MG cells were seeded in 1500 cells/well in a 96-well plate and cultured for 24 h to enable cell attachment. The test compounds (RAD114, RAD118, RAD119, RAD120 at 0.01 to 100 μM; AqB011 at 0.03 to 100 μM; or DMSO at 0.0001 to 1%) in cell culture medium were added to the cells. After an incubation period of 72 h, the cell culture medium was removed from the plate and 50 μL/well of the colorimetric WST-8 reagent, diluted 1:10 in the respective cell culture medium, was added to the cells. After 1 h incubation at 37 °C, the plate was placed into the Multiskan FC Microplate Photometer (Thermo Fisher Scientific, Waltham, MA) and measured at a wavelength of 450 nm.

### Spheroid invasion assay

U87-MG cells were seeded in round-bottom 96-well plates (25,000 cells/well) and incubated for 24 h at 37 °C to induce spheroid formation. The VitroGel® Hydrogel Matrix solution (TheWell Bioscience, Monmouth Junction, NJ) was mixed 2:1 (v/v) with cell culture medium (DMEM + 30% FBS) containing the respective test compounds (DMSO, RAD114, RAD118, RAD119, RAD120) in 3-fold concentration (300 µM). After the supernatant was carefully removed from the spheroid plate, the hydrogel mixture was added to the spheroids and incubated for 20 min at room temperature. The consolidated hydrogel was covered with cell culture medium (DMEM + 10% FBS) containing the respective compounds at 100 µM and incubated at 37 °C to complete the polymerization process. Images were taken every 6 h for 6 days using the Incucyte® SX5 Live-Cell Analysis System (Sartorius, Göttingen, Germany).

### Generation of tumor xenografts and xenograft tissue slice tandem co-cultures

Immunodeficient NSG mice (NOD/SCID/IL-2Rγ-null mice; Charles River Laboratories, Sulzfeld, Germany) were maintained at standard conditions and allowed access to lab food and water *ad libitum*. All mouse studies were approved by the responsible research ethics committee and the local authorities (TVV 59/20 and TVV23/24, Saxony Regional Directorate) and carried out according to the national regulations of animal welfare and the national legislation on the use of animals for research (Animal Welfare Act, Animal Welfare Experimental Animal Ordinance). Work complied with the ARRIVE guidelines and the EU Directive 2010/63/EU for animal experiments, including strict and careful consideration of the 3R principles, measures to reduce animal suffering, and the definition of humane endpoints and euthanasia methods.

For tumor xenograft generation, G55T2 (2.5×10^6^ cells/150 μL) or U87-MG cells (5×10^6^ cells/150 μL) were diluted in PBS and injected into both flanks of 3- to 6-month-old NSG mice. After two to three weeks, when the formed xenograft tumors reached a size of ~10 mm in diameter, mice were euthanized, and the tumors were excised under aseptic conditions and embedded in warm 4% agarose. After the agarose had solidified, the embedded tumor was cut into 300 µm-thick slices using a Leica VT1200S Vibratome (Leica Microsystems, Nussloch, Germany). The tumor slices were collected in cold PBS.

For the generation of xenograft tissue slice tandem co-cultures (XTCCs), brain tissue slices from 3- to 4-month-old NSG mice were prepared using established protocols [24, 25]. Briefly, a 6-well tissue culture (TC) plate was filled with 1 mL/well RPMI 1640 cell culture medium (Gibco, Thermo Fisher Scientific, Carlsbad, CA) supplemented with 10% FBS and 1% Penicillin-Streptomycin. The plate was loaded with TC inserts for 6-well plates, (PET, transparent, pore size: 0.4 µm, Sarstedt, Nümbrecht, Germany) and placed at 37 °C to pre-warm the medium. The mouse brain was surgically resected under aseptic conditions and embedded in warm 4% agarose. The solidified agarose block was cut into 300 µm-thick cortex slices using a Leica VT1200S Vibratome (Leica Microsystems). The brain slices were immersed in cold PBS and immediately placed on the membrane of the trans-well inserts (Sarstedt) to create an air-liquid-interface (ALI) cultivation environment.

The tumor xenograft slices were placed under a stereomicroscope and punched out with a 1.5 mm diameter biopsy stamp, prior to being transferred on top of the 300 µm-thick cortex slices using two sterile spatulas. This allowed for selecting tumor areas with comparable phenotype and in particular for excluding larger necrotic areas, and ensured that all samples were exactly equal in size. The co-culture was incubated at 37 ⁰C for 24 h to allow the tumor xenograft punch to grow on the cortical tissue before the test compounds were applied. For excluding intertumor heterogeneity, a single tumor xenograft was employed within each experiment, allowing for the direct comparison between different treatments within an experiment. Repetition experiments based on different tumor xenografts allowed for monitoring intertumor heterogeneities, while the use of multiple punches from a given tumor xenograft slice within the same treatment group also revealed intratumor heterogeneity.

### GBM xenograft tissue slice tandem co-culture cultivation and processing

The culture medium of the 6-well XTCC plate was replaced with fresh medium containing the respective test compounds or vehicle control (DMSO) 24 h after co-culture preparation (day 1). The medium was replaced every other day over a total incubation time of 8 days. After tissue fixation with 4.5% formaldehyde (ROTI®Histofix, Carl Roth, Karlsruhe, Germany) for 24 h and washing with PBS, the co-cultures were coated with a warm matrix containing 2% agarose and 2.5% gelatine to protect the fragile constructs physically during tissue dehydration. The embedded co-cultures were placed in tissue cassettes, stored in 70% Ethanol for 24 h, and placed in an automated tissue processor (Citadel 1000, Shandon/Thermo Fisher Scientific). Tissue dehydration was conducted in 70% Ethanol (60 min), 80% Ethanol (30 min), 96% Ethanol (30 min), Isopropanol (2×45 min), Xylol (2× 30 min), and Paraffin (2× 90 min; Pure™ Paraffin, MEDITE Medical, Burgdorf, Germany). Subsequently, the tissues were embedded in Paraffin (Tissue-Tek Embedding Console Model 4715, Sakura, Japan), cut into 6 µm-thick slices using a Leica Reichert-Jung Biocut 2035 microtome (Leica Biosystems), and collected on histological Polysine-coated adhesion glass slides (Epredia™ Polysine Adhesion Slides, Gerhard Menzel, Braunschweig, Germany). The glass slides were placed on a Leica Histoplate stretching table (Leica Biosystems) heated to 39 ⁰C to dry and stretch the Formalin-fixed Paraffin-embedded (FFPE) tissue sections before immunohistochemical staining.

### Immunohistochemistry

Microscopic slides were kept at 58 ⁰C overnight, heated in the microwave for 5 min at 700 W, and incubated twice in Neo-Clear™ Xylene Substitute solution (Merck, Darmstadt, Germany) for 10 min. After immersing the slides through a decreasing alcohol series (100%, 96%, 70% Ethanol), they were washed for 1 min in distilled water and 5 min in 1× PBS-Tween (PBS-T). For antigen retrieval, the slides were kept at 88–95 °C in 1× Sodium citrate buffer, pH 6 (for Vimentin- and phospho-p44/42 MAPK staining) or 1× EDTA buffer, pH 8 (for Ki-67 staining) in the microwave for 15 min at 900 W. The slides were allowed to cool down for 10 min at room temperature, washed again for 5 min in 1× PBS-T, and incubated in 0.3% Hydrogen peroxide (H_2_O_2_) diluted in PBS-T for 30 min at 4 ⁰C to block endogenous peroxidases. After washing three times for 5 min in 1× PBS-T, a Liquid Barrier Marker (Pap Pen, Merck) was applied around the tissue sections and non-specific binding sites were blocked by incubation in blocking solution (PBS-T + 2% Bovine Serum Albumin (BSA)) with 10% Normal Horse Serum or 10% Normal Goat Serum (both from Vector Laboratories, Burlingame, CA) for 1 h at room temperature. After washing three times for 5 min each in 1× PBS-T, the slides were incubated overnight at 4 °C with the primary antibody (monoclonal mouse anti-Vimentin clone V9 (M0725), 1:1000; monoclonal mouse anti-human Ki-67 clone MIB-1 (M7240), 1:100; both from DakoCytomation, Glostrup, Denmark; polyclonal rabbit anti-active Caspase-3, 1:50, Enzo Life Sciences, Lörrach, Germany; rabbit monoclonal anti-phospho-p44/42 MAPK (4370S), Cell Signalling Technology, Danvers, MA, 1:500), diluted in blocking solution. Negative control experiments were conducted without the primary antibody. On the next day, the slides were washed three times for 5 min in 1× PBS-T and incubated with the secondary antibody (biotinylated horse anti-mouse IgG or biotinylated goat anti-rabbit IgG, both from Vector Laboratories) diluted in blocking solution for 1 h at room temperature. The slides were washed three times for 5 min in 1× PBS-T and incubated with avidin/biotin complex (ABC) reagent (Vectastain® Elite® ABC-HRP Kit, Vector Laboratories) diluted in PBS-T for 1 h at room temperature. After a final three-times washing step for 5 min in 1× PBS-T, the staining solution (3,3’-Diaminobenzidine tetrahydrochloride (0.1% DAB) with 0.003% H_2_O_2_; both from Carl Roth) was prepared and the slides were incubated in the staining solution until the brown staining could be observed under the microscope (Axioscope, Carl Zeiss, Oberkochen, Germany). The reaction was stopped by immersing the slides in distilled H_2_O, and nuclei were stained for 30 s with Hematoxylin solution according to Gill II (Carl Roth). Excess Hematoxylin solution was removed by swiveling the slides for 5 min in distilled H_2_O. After dehydration through an increasing alcohol series (70% Ethanol, 96% Ethanol, 100% Ethanol), the slides were incubated for 5 min in n-Butyl acetate (Carl Roth), mounted with mounting medium (Entellan™, Merck), cover-slipped and allowed to dry overnight at room temperature. Microscopic pictures were taken with the All-in-One Fluorescence Microscope BZ-X810 (Keyence, Itasca, IL). Quantification of the staining was performed with the Image J software.

For the determination of total tumor area and tumor invasion area, the tumor upper and lower area (i.e., the tumor area above and below the horizon of the normal brain slice, respectively), as well as the total area of invading cells were determined as described previously [24, 25]. To ensure direct comparability, the calculation of cell invasion is normalized to tumor size in order to control for any differences in tumor size despite the prior punching.

### Annexin V apoptosis assay and flow cytometry-based cell cycle analysis

G55T2 and U87-MG cells were seeded in a 12-well plate (G55T2: 0.03×10^6^ cells/well, U87‑MG: 0.04×10^6^ cells/well) and cultured overnight at 37 ⁰C, 5% CO_2_. On the next day, the cells were treated with the respective compounds (RAD114, RAD118, RAD119, RAD120) and DMSO control for 48 h in total. Prior to flow cytometry analysis for apoptosis measurement, cells were trypsinized, transferred to Eppendorf tubes, and centrifuged for 5 min at 1000 × g. The supernatant was removed, and Annexin V-Alexa 647 (Thermo Fisher Scientific) diluted in Annexin Binding Buffer (10 mM HEPES, pH 7.4, 140 mM NaCl, 2.5 mM CaCl_2_) was added to the cells. The cells were incubated for 15 min in the dark at room temperature. Then, Propidium iodide (PI) solution was added in a final concentration of 20 μg/mL, and cells were incubated for 5 min in the dark at room temperature. Apoptotic cells were detected in the RL1 channel, and necrotic cells in the BL2 channel using an Applied Biosystems™ Attune™ Acoustic Focusing Cytometer (Thermo Fisher Scientific). Analysis was conducted with the Attune software v2.1.0.

For cell cycle analysis, cells were treated as above. To arrest cells in the G2/M phase, Nocodazole (G55T2: 25 ng/mL, U87: 50 ng/mL; Carl Roth) was added to the cells for 18 h (G55T2) or 22 h (U87‑MG) before fixation. For flow cytometry measurement, cells were trypsinized, transferred to Eppendorf tubes, and centrifuged for 5 min at 1000 × g. The supernatant was removed, and cells were fixed with 70% cold Ethanol for 30 min at − 20 ⁰C. Then, cells were centrifuged for 5 min at 1000 × g, the supernatant was removed, and cells were resuspended with 50 μg/mL RNase A diluted in PBS. For RNase digestion, cells were incubated at 37 ⁰C for 1 h. Propidium iodide (PI) solution was added to achieve a final concentration of 20 μg/mL, and cells were incubated for 5 min in the dark at room temperature. Cell cycle phases were detected in the BL2 channel using an Applied Biosystems™ Attune™ Acoustic Focusing Cytometer (Thermo Fisher Scientific). Cell cycle analysis was conducted with the Attune software v2.1.0.

### Quantitative real time PCR (RT-qPCR)

For RT-qPCR, total RNA was extracted from cell lysates and homogenised tumor xenografts using TRIfast Reagent (VWR, Darmstadt, Germany), as described in the LogSpin protocol. After cDNA synthesis with the RevertAid H Minus FirstStrand cDNA Synthesis Kit (Thermo Fisher Scientific), RT-qPCR was performed using the PerfeCTa® SYBR® Green FastMix® ROX (QuantaBio, Hilden, Germany), primer mix, and cDNA (1:10 dilution). Using the StepOnePlus Real Time System, pre-incubation was performed at 95 °C for 2 min, followed by 50 amplification cycles (95 °C for 10 s, 55 °C for 10 s, and 72 °C for 10 s). The ΔΔCt method was used to calculate mRNA levels relative to the reference genes *hActin* and *RPLP0*.

### Statistics

Statistical analyses were performed with GraphPad Prism software Version 10.1.0 (San Diego CA). The data are presented as mean ± standard deviation (SD). Statistical differences were determined with ordinary one-way ANOVA for equal variances and Welch’s ANOVA for unequal variances. Dunnett’s multiple comparison post-hoc test was used for pairwise comparisons, represented by asterisks as *, p < 0.05; **, p < 0.01; ***, p < 0.001 and ****, p < 0.0001, or ns for not significant differences compared to the DMSO vehicle control.

## Results

### Semisynthetic indole analogs show little dose-dependent cytotoxicity

Potential long-term cytotoxic effects in G55T2 and U87-MG cell lines were assessed after 72 h treatment with the different indole compounds at concentrations up to 100 µM, using a WST‑8 assay for metabolic activity. In U87-MG cells, no effects were observed up to 30 µM. At 100 µM, some growth inhibition was observed for U87-MG cells, which, however, was at least in part attributable to the DMSO content as indicated by the comparison with the DMSO vehicle control (Suppl. Figure 2, left). In contrast, no solvent effects were observed in G55T2 cells. Cytotoxicity was found only at the highest concentration of RAD118, RAD119, and RAD120 (100 µM), with some tumor cell-inhibitory effects of RAD120 also evident at 10 µM (Suppl. Figure 2, right). Taken together, 2D cell culture revealed overall little effect of the RAD compounds on cell viability.

### Semisynthetic indole analogs inhibit GBM invasion in spheroids and scratch assays *in vitro*, and in *ex vivo* xenograft tissue slice tandem co-cultures

**Fig. 1:**
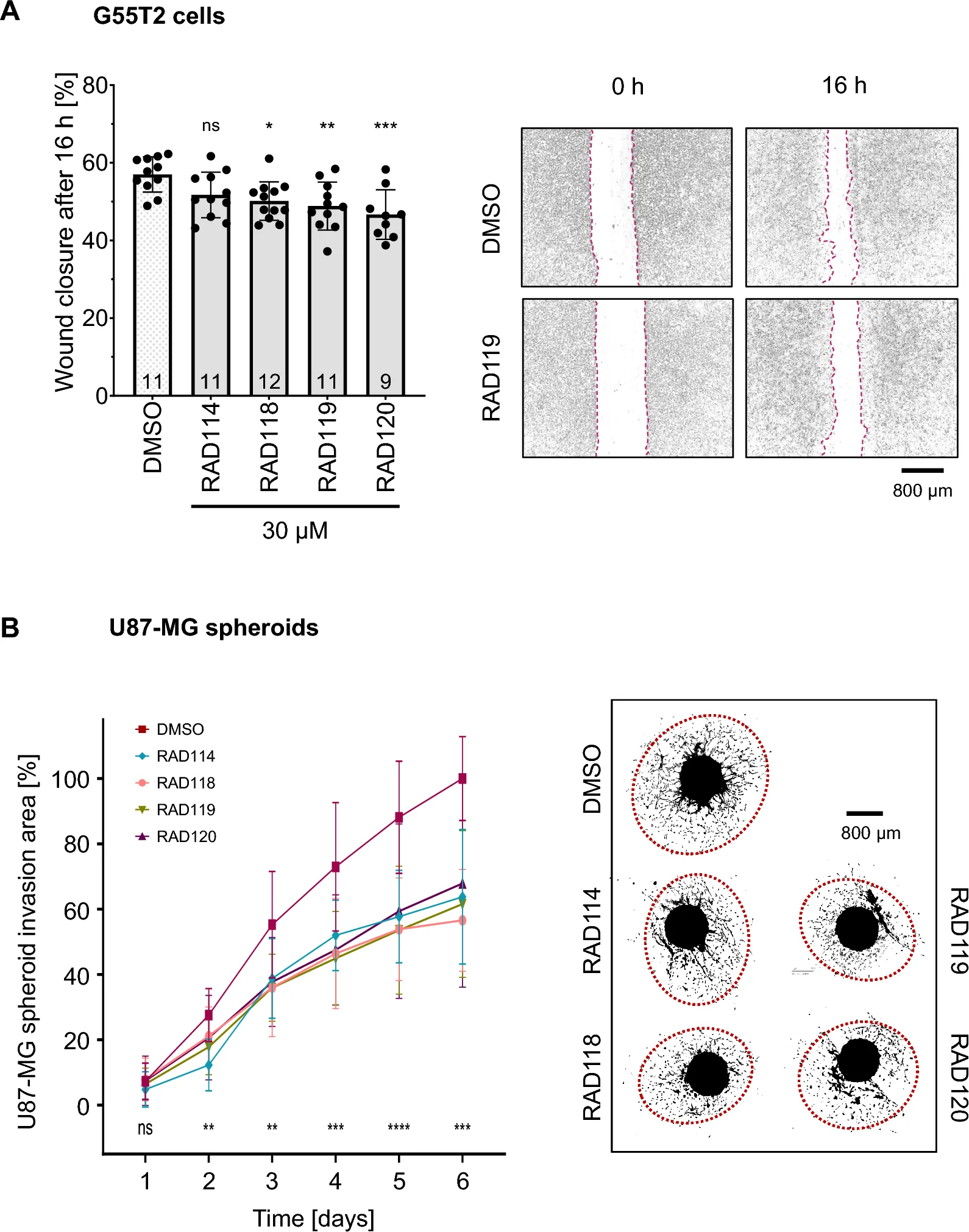
Effects of the indole analogs in 2D or 3D *in vitro* cell culture. (**A**) Quantification and representative images of G55T2 wound-closure migration after 16 h of drug treatment (30 μM indole alkaloids, 0.3% DMSO). Data were compiled from three biological replicates. The numbers of technical replicates are shown above the x-axis. Statistically significant differences between treatments and vehicle control were determined with one-way ANOVA using Dunnett’s multiple comparisons test, ns = not significant. Asterisks indicate statistically significant differences (*, p < 0.05; **, p < 0.01; ***, p < 0.001 and ****, p < 0.0001). (**B**) Quantification and representative images of U87-MG spheroid invasion after 6 days of compound treatment (100 μM indole compounds, 1% DMSO). Results are from three biological experiments with 9 to 17 technical replicates. Statistically significant differences between treatments and vehicle control were determined with one-way ANOVA using Dunnett’s multiple comparisons test, ns = not significant. Asterisks indicate statistically significant differences (*, p < 0.05; **, p < 0.01; ***, p < 0.001 and ****, p < 0.0001)

Effects of the indole compounds on cell migration and spheroid invasiveness were first evaluated *in vitro* by imaging the lateral movement of G55T2 cells into a scratch wound area, and by monitoring the dispersion of motile U87-MG spheroid cells into the surrounding hydrogel matrix over time (Fig. 1). For scratch assays, G55T2 cells were treated with 30 µM concentrations of compounds. RAD114 did not affect G55T2 lateral cell migration, whereas the indole alkaloid analogs RAD118, RAD119 and RAD120 reduced cell migration to a small but significant extent (Fig. 1A). In contrast, all tested indole compounds reduced U87-MG cell outgrowth from spheroids by approximately 40% as compared to vehicle control, indicating a robust block of cell migration into the gel (Fig. 1B**,** with digital post-processing into black & white for better visibility). To ensure above-threshold exposures of the tumor cells to the test compounds in the hydrogel and spheroid mass, the U87-MG spheroids were treated with 100 µM doses.

**Fig. 2:**
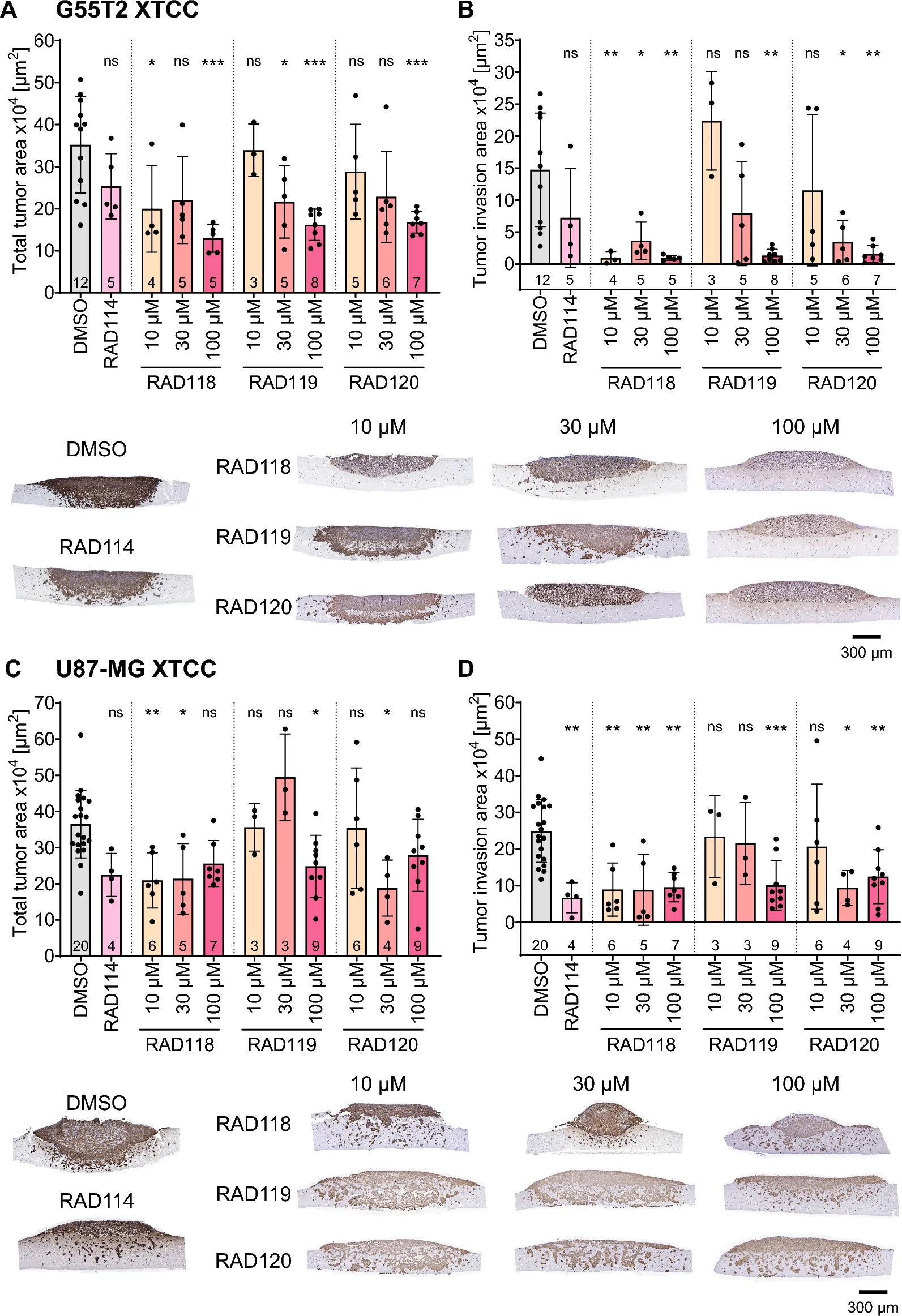
Effects of the indole analogs in XTCCs. Compiled data in swarm plots (mean ± SD) show results from quantification of the (**A, C**) total tumor area and (**B, D**) tumor invasion area of (**A, B**) G55T2 and (**C, D**) U87-MG xenograft tissue in slice tandem co-cultures (XTCCs), treated with vehicle control (1% DMSO), RAD114 (100 μM), or RAD118, RAD119, or RAD120 (10, 30, 100 μM). Representative microscopic pictures of Vimentin-stained XTCCs are shown below the respective graphs. Data were compiled from at least two biological replicates. The numbers of technical replicates are shown above the x-axis. Statistically significant differences between treatments and vehicle control were determined with ordinary one-way ANOVA for equal variances and Welch’s ANOVA for unequal variances using Dunnett’s multiple comparisons test, ns = not significant. Asterisks indicate statistically significant differences (*, p < 0.05; **, p < 0.01; ***, p < 0.001 and ****, p < 0.0001)

Based on the promising *in vitro* findings described here and published previously [21]*,* we further explored their efficacy using a more complex system closer to an *in vivo* situation. For this, the *ex vivo* GBM xenograft tissue slice tandem co-culture (XTCC) models with G55T2 and U87-MG tumor xenografts were employed [24, 25]. GBM XTCCs were treated with RAD114 at 100 μM or its analogs RAD118, RAD119, and RAD120 at 10, 30, and 100 μM for 8 days, and assessed for tumor growth and invasion as compared to the 1% DMSO vehicle control (Fig. 2). At day 8, Vimentin-stained FFPE sections were microscopically analyzed for the total tumor area, i.e., the bulk tumor mass above or in the normal brain host tissue of the tandem tissue slice culture, and the tumor invasion area, i.e., the extent of cells invading into the normal brain host tissue outside the bulk tumor mass. At 100 µM, the parent compound RAD114 had moderate effects on reducing the total tumor area in G55T2 XTCCs (Fig. 2A), but caused a ~50% reduction of the tumor invasion area (Fig. 2B). The semi-synthetic analogs induced dose-dependent decreases in both total tumor area and tumor invasion area in G55T2 XTCCs. In reducing tumor invasion area, all three RAD inhibitors showed dose-dependent efficacy, with RAD118 emerging as the most potent (effective at 10 µM), and RAD119 and RAD120 effective at 30 µM. Almost complete inhibition of tumor invasion in XTCCs was seen for RAD118 at all doses tested, and for RAD119 and RAD120 at higher concentrations (Fig. 2B).

This selective action of the RAD compounds on inhibiting tumor cell invasion rather than bulk tumor growth was also seen in U87-MG XTCCs. The parent compound RAD114 showed ~40% reduction of the total tumor area similar to effects described in G55T2 (above), whereas a substantial decrease in tumor invasion area achieved ~70% reduction (Fig. 2C and D). Again, RAD118 was most efficacious of the indole analogs, effective at 10 µM. The invasion inhibitory effects are also summarized in Table 1.

**Table 1:** Percentage inhibition of GBM invasiveness by the indole parent compound (RAD114) at 100 µM, the semisynthetic derivatives (RAD118, RAD119, RAD120) at 10, 30, and 100 µM, and the known AQP1 ion channel blocker AqB011 at 10, 30, and 100 µM, tested in xenograft tissue slice tandem co-cultures

**G55T2 XXTC**		**RAD114**	**RAD118**		**RAD119**	**RAD120**	**AqB011**
**10 μM**		-	94%		ND	22%	46%
**30 μM**		-	75%		46%	77%	69%
**100 μM**		51%	94%		91%	89%	90%

**U87-MG XXTC**		**RAD114**	**RAD118**		**RAD119**	**RAD120**	**AqB011**
**10 μM**		-	64%		ND	ND	29%
**30 μM**		-	65%		14%	62%	45%
**100 μM**		73%	62%		60%	50%	54%

Percent values are standardized to the 1% DMSO vehicle control. ND = not detectable

This selective action of the RAD compounds on inhibiting tumor cell invasion rather than bulk tumor growth was also seen in U87-MG XTCCs. The parent compound RAD114 showed ~40% reduction of the total tumor area similar to effects described in G55T2 (above), whereas a substantial decrease in tumor invasion area achieved ~70% reduction (Fig. 2C, D). Again, RAD118 was the most efficacious of the indole analogs, effective at 10 µM. The invasion inhibitory effects are also summarized in Table 1.

### The semisynthetic indole analogs exert only marginal effects on apoptosis and cell-cycle

**Fig. 3:**
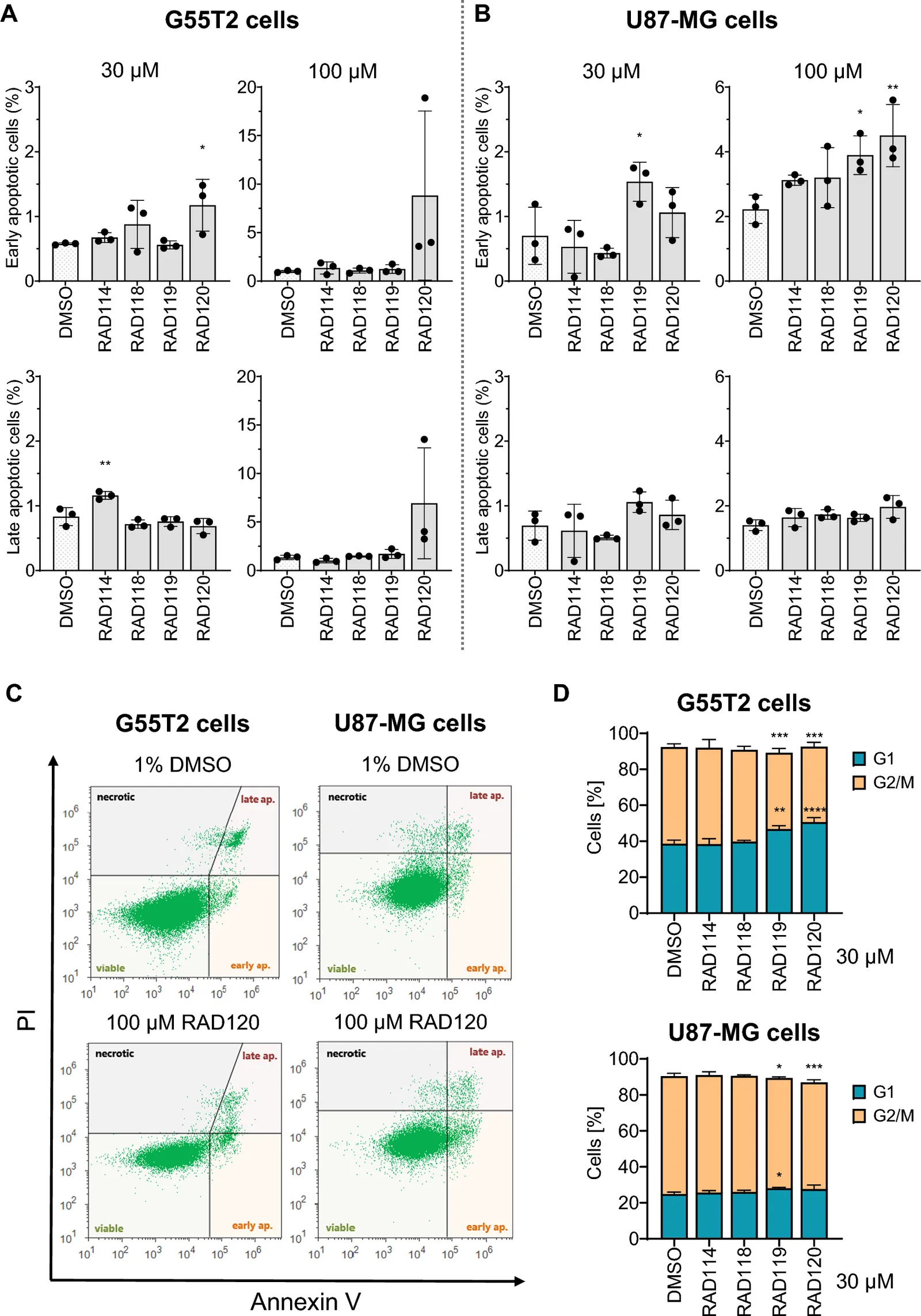
Analysis of apoptosis and cell cycle alterations upon treatment with the indole analogs. Quantification of early and late apoptosis in (**A**) G55T2 and (**B**) U87-MG cells after 48 h of compound treatment at 30 and 100 μM measured by flow cytometry. Results are from one biological experiment with 3 technical replicates. Statistically significant differences between each treatment and DMSO were determined with one-way ANOVA using Dunnett’s multiple comparisons test. (**C**) Representative examples of gated cell populations treated with 1% DMSO or 100 μM RAD120. (**D**) Compiled data in stacked bar graphs (mean ± SD) showing the quantification of percentage cells in the G1 (blue) and G2/M phase (orange) after 48 h treatment with the test compounds (0.3% DMSO, 30 μM RAD114, RAD118, RAD119, RAD120). Results are from one biological experiment with 3 technical replicates. Statistical differences between each treatment and DMSO were determined with one-way ANOVA using Dunnett’s multiple comparisons test

To further study possible underlying cellular mechanisms of the inhibition of tumor cell invasion, we analyzed potential apoptosis-inducing and cell-cycle-dependent effects of the indole compounds. Flow cytometry-based Annexin V Apoptosis assays were performed in G55T2 and U87-MG cells (Fig. 3). In both cell lines, some slight but overall minor increases in early apoptosis were observed, whereas the percentage of late apoptotic cells remained largely unchanged (Fig. 3A–C). Effects were mostly seen in the case of RAD120 treatment.

For the assessment of alterations in cell cycle, the cells were treated with the test compounds for 48 h and then arrested in the G2/M phase using Nocodazole for 18 h (G55T2) or 22 h (U87‑MG) before fixation. The cells were then stained with Propidium iodide (PI) solution, and cell cycle phases were detected in the BL2 channel. The direct comparison of cells ± Nocodazole confirmed that the cells were efficiently arrested when they reached G2/M, leading to a higher percentage of cells in the G2/M phase after treatment with Nocodazole (data not shown). Importantly, this allowed for analyzing alterations in the cell cycle progression rate under compound treatment, by comparing the percentage of cells reaching the Nocodazole-mediated G2/M block in the given time period. In G55T2 cells, RAD119 and RAD120, but not RAD114 or RAD118, led to a slightly increased percentage of cells in the G1 phase with fewer cells reaching the G2/M phase (Fig. 3D). While this indicated that these compounds slightly slowed down the cell cycle, the effects of compounds were minor. Likewise, in U87-MG cells, essentially no difference was seen. Taken together, this demonstrated that the invasion-inhibitory effects of the indole alkaloid analogs are not mediated by altering the cell cycle or inducing apoptosis.

### The sulfonamide AqB011 reduces GBM invasion in xenograft tissue slice tandem co-cultures

**Fig. 4:**
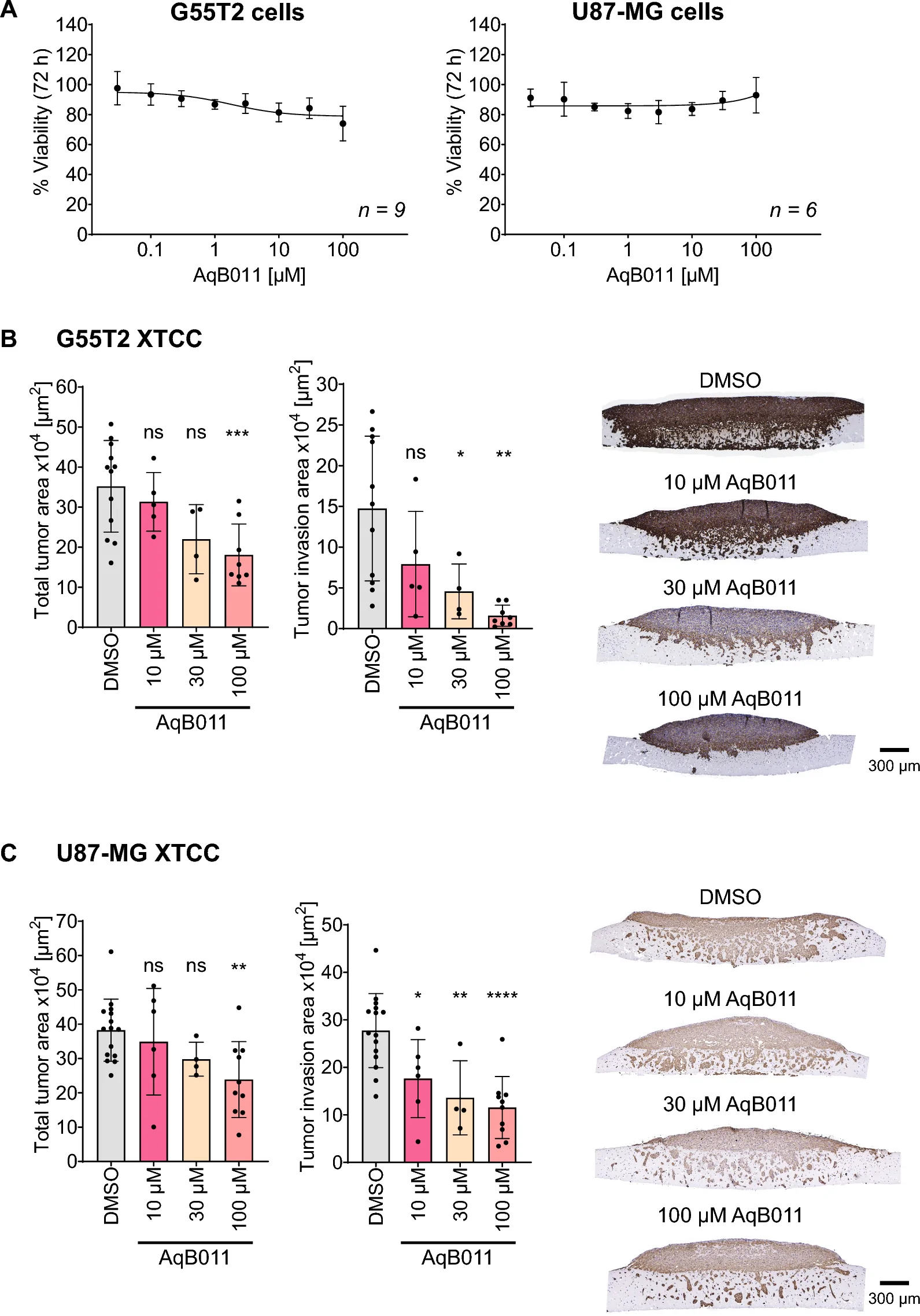
Effects of the aquaporin-1 inhibitor AqB011. (**A**) AqB011 dose-response curves (0.03–100 μM) normalized to DMSO-treated controls measured with WST-8 after 72 h of incubation using G55T2 and U87-MG cells. Results are from at least two biological replicates with 6 to 9 technical replicates. (**B**) Compiled data in swarm plots (mean ± SD) show the quantification of the total tumor area (left) and tumor invasion area (right) of G55T2 XTCCs treated with vehicle control (1% DMSO), or AqB011 (10, 30, 100 μM). (**C**) Compiled data in swarm plots (mean ± SD) show the quantification of the total tumor area (left) and tumor invasion area (right) of U87-MG XTCCs treated with vehicle control (1% DMSO), or AqB011 (10, 30, 100 μM). Rightmost panels in (**B**) and (**C**): representative microscopic pictures of Vimentin-stained XTCCs. Data were compiled from at least two biological replicates with 4 to 15 technical replicates. Statistically significant differences between treatments and vehicle control were determined with ordinary one-way ANOVA for equal variances and Welch’s ANOVA for unequal variances using Dunnett’s multiple comparisons test, ns = not significant. Asterisks indicate statistically significant differences (*, p < 0.05; **, p < 0.01; ***, p < 0.001 and ****, p < 0.0001)

In addition to the indole compounds, another promising agent, AqB011, was tested for the first time in the XTCC model (Fig. 4). AqB011 belongs to the class of sulfonamides that has been previously reported to inhibit AQP1 and to reduce the migration and invasion of cancer cells *in vitro* [26, 27]. Expression of AQP1 was confirmed by RT-qPCR in both cell lines as well as in the cell line-derived xenograft tumors, with the latter revealing substantially higher expression levels (Suppl. Figure 3A, B). Initially, the dose-dependent effects of AqB011 on cell viability were assessed in G55T2 and U87-MG cells using WST‑8 assay (Fig. 4A). After compound treatment in cell culture for 72 h, no substantial effects of AqB011 on viability were observed at any dose tested for G55T2 or U87-MG cells.

In contrast, AqB011 caused moderate dose-dependent reductions in total tumor area, and strong dose-dependent reductions in invasiveness in G55T2 (Fig. 4B) and U87-MG (Fig. 4C) cells. GBM XTCCs revealed a potent inhibition of invasiveness (Fig. 4B). More specifically, an only up to ~50% reduction of total tumor area was seen at the highest dose, indicating moderate inhibitory effects of AqB011 on the growth of the bulk tumor mass, while the analysis of the tumor invasion areas revealed an almost complete abolishment of the invasive capacity of the tumor cells in G55T2 XTCCs. Notably, in both GBM types, 10 µM was already found sufficient for markedly inhibiting tumor cell infiltration of the normal brain tissue, with a profound, albeit statistically not significant ~50% reduction in G55T2 XTCCs and a statistically significant >35% decrease in U87-MG XTCCs (Fig. 4B, C). Thus, direct comparisons of effects on total tumor area with tumor invasion area revealed greater magnitudes of effects on the invasive potential, showing a stronger effect of AqB011 on inhibiting motility as compared to bulk tumor growth.

### Invasion inhibitory effects of the semisynthetic indole analogs and AqB011 are not based on cell proliferation and apoptosis

When analyzing XTCC sections for cell markers of proliferation or apoptosis, no major effects of compounds were observed. More specifically, immunohistochemical staining for the proliferation marker Ki-67 revealed a broader distribution of Ki-67 total counts between the samples. A slight, but not statistically significant trend towards decreased cell proliferation was seen upon RAD120 treatment of G55T2 XTCCs, but not in their U87-MG counterparts (Suppl. Figure 4A, B). Likewise, no effects were seen in the numbers of apoptotic cells, as determined by active Caspase−3 staining (Suppl. Figure 4C, D). This extends the *in vitro* findings (see Fig. 3 above) to confirm that these cell lines also displayed no major alterations in apoptosis or cell cycle distribution in the context of a native tissue environment. This finding thus also highlights the interpretation that the compounds are preferentially active in inhibiting invasion rather than compromising cellular mechanisms involving in maintaining viability.

As an example of vital cellular mechanisms which are also relevant in the oncological context and may be related to invasion, we selected the MAPK/ERK pathway which transmits signals from growth factor receptors to the nucleus and stained selected XTCC samples for phospho-p42/44 (i.e., activated p42/44). U87-MG XTCCs revealed quite profound staining which was in part cytoplasmic but, as to be expected, mainly nuclear. Invading cells showed a tendency towards stronger signals as compared to the bulk tumor mass. Treatment with AqB011 showed a dose-dependent, overall reduced staining intensity at 100 µM which, however, may also be attributed to the decrease in invading cells. A trend towards lesser staining was also observed in the case of RAD119 treatment (Suppl. Figure 5). While in G55T2 XTCCs the overall staining intensity was weaker, the tendency towards stronger signals in invading cells was observed as well. Again, a reduction in their number and staining intensity was observed upon compound treatment (Suppl. Figure 6). The sample size, however, did not allow for quantitation and highlights the need of sufficient XTCC samples, especially when considering the comparatively small size of the sections. Also, for drawing a causal link e.g. between MAPK/ERK inhibition and the reduced invasive phenotype, additional experiments would be required.

### Enhancement of anti-invasive properties by combinations of compounds

Both the indolamine and the sulfonamide derivatives, though quite different in chemical structures, showed profound anti-invasive properties that prompted us to test these compounds in combination. We deliberately selected sub-optimal concentrations for assessing effects of treatment combinations. Additionally, we included Temozolomide (TMZ) given its use as the standard cytostatic drug in glioblastoma treatment, again at a dosage previously determined to be sub-therapeutic in this assay [25].

**Fig. 5:**
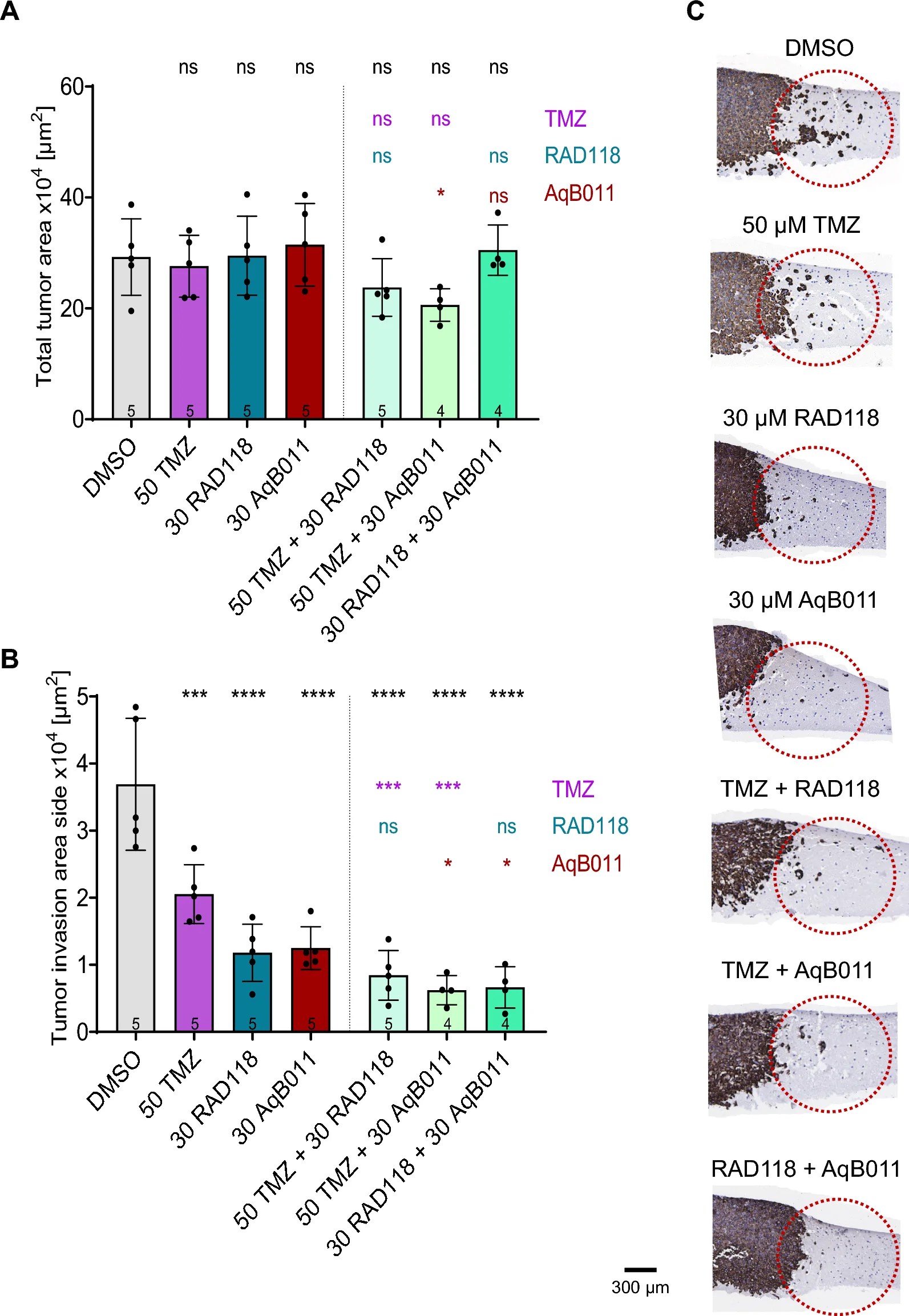
Effects of the indole analog RAD118, the standard cytostatic Temozolomide (TMZ) and AqB011, alone or in combination, in G55T2 XTCCs. Compiled data in swarm plots (mean ± SD) show the quantification of the (**A**) total tumor area and (**B**) tumor invasion area (lateral) after treatment with sub-optimal compound concentrations. (**C**) Representative sections of microscopic immunohistochemistry images showing lateral tumor cell invasion. Red circles highlight the invasive zones of interest. Statistically significant differences between treatments and the DMSO vehicle control were determined with one-way ANOVA using Dunnett’s multiple comparisons test and are shown in black asterisks (*, p < 0.05; **, p < 0.01; ***, p < 0.001 and ****, p < 0.0001), ns = not significant. Statistically significant differences between the combination treatments and the respective single treatments (TMZ, RAD118, AqB011) were determined with one-way ANOVA using Dunnett’s multiple comparisons test and are shown in the respective color of the bar of the single treatments (*, p < 0.05; **, p < 0.01; ***, p < 0.001 and ****, p < 0.0001), ns = not significant

The analyses of total tumor areas in G55T2-based XTCCs after single or combined treatments showed that the concentrations chosen for the selected compounds (TMZ, RAD118, and AqB011) individually were insufficient for inhibiting bulk tumor growth (Fig. 5A**,** left). The combination of TMZ with AqB011 showed slight reduction of the tumor mass, but only when compared with AqB011 alone (Fig. 5A**,** right). In contrast, the single compounds caused pronounced decreases in tumor cell invasion (Fig. 5B**,** left). Notably, the combination of the compounds RAD118 and AqB011 with low-dose TMZ enhanced the inhibition of tumor cell invasion. Inhibition of tumor cell invasion by > 80% was also observed with the combination of low doses of RAD118 and AqB011 (Fig. 5B**,** right). Representative sections of microscopic immunohistochemistry images showing lateral tumor cell invasion after each treatment are shown in Fig. 5C.

### SwissADME predicts likeliness of BBB permeability of all RAD compounds

The open access SwissADME software [28] was used to calculate the *in silico* physicochemical parameters for all RAD compounds tested in this study and AqB011. The data were compared to Lipinski’s drug-like ‘‘Rule of Five’’ (MW < 500; HBA < 10; HBD < 5; cLog P < 5), which are parameters that serve as guidelines for drug-likeness [29]. The physicochemical parameter profiles (i.e. MW, HBA, HBD, cLogP) for these compounds are shown in Table 2. Of note, all compounds tested in these studies have drug-like parameters with no Lipinski violations. Furthermore, RAD114, RAD118, RAD119, RAD120, and AqB011 were profiled using SwissADME to determine if this software deemed these compounds likely to be blood brain barrier (BBB) permeable. Of note, all RAD compounds were predicted to be BBB permeable whilst AqB011 was not.

**Table 2:** Physiochemical parameter data and number of Lipinski “Rule of Five” violations for RAD114, RAD118-RAD120, and AqB011 identified for each compound *in silico*

**Compound**	**Physicochemical parameters** ^a^				**No. of Lipinski “Rule of Five” violations**
	**MW**	**cLog*P***	**HBA**	**HBD**	
**RAD114**	161	2.13	2	2	0
**RAD118**	294	3.12	2	2	0
**RAD119**	294	3.12	2	2	0
**RAD120**	250	3.08	2	1	0
**AqB011**	454	2.85	6	3	0

^a^*In silico* calculations performed using SwissADME, MW = molecular weight (Da); cLogP = calculated partition coefficient; HBA = H-bond acceptors, HBD = H-bond donors

## Discussion

In this study, we tested for the first time the effects of novel indole compounds (RAD114, RAD118, RAD119, RAD120) and the sulfonamide AqB011 previously reported to inhibit AQP1 on cell invasion in *ex vivo* xenograft tissue slice tandem co-culture (XTCC) models. The compounds substantially reduced the invasiveness of G55T2 and U87-MG tumor xenografts into adjacent normal brain tissue at concentrations ranging from 10 to 100 µM, depending on the cell line and compound, with lesser effects on bulk tumor growth. These results support our previous *in vitro* results [21] which showed that cancer cell invasion can be specifically restrained by targeting water and ion channels without cytotoxic side effects, revealing a novel and promising potential treatment option for highly invasive GBMs.

This conclusion was also supported by the minimal effects seen for the compounds on tumor cell proliferation and apoptosis, suggesting the primary mode of action of the compounds was inhibition of cellular mechanisms involved in tumor cell infiltration into the normal brain tissue.

The indole compounds have been shown previously to reduce GBM invasion *in vitro* and to block AQP1 water channels in the *Xenopus laevis* oocyte expression system [21]. To set the stage for combinational testing approaches, an agent from another compound class previously shown to reduce cancer cell migration *in vitro* [26, 27], the aryl sulfonamide AqB011, was included in the current analysis as well. AqB011 has previously been reported to inhibit AQP1 [30]. The dual water and ion channel AQP1 has been shown to be upregulated in GBM and has been associated with cell migration and invasion of multiple cancers, including colon cancer [31, 32], lung cancer [33, 34], endothelial cancer [35] and GBM [36, 37], as well as neural crest cell migration during nervous system early development [38]. Moreover, blocking of AQP1 restrained tumor growth and invasion in several studies [26, 27, 39–41]. Thus, we assume that AQP1 water and ion channels are involved in the complex interplay of factors promoting tumor progression. Still, the direct proof that the anti-invasive effect of AqB011 is indeed mediated through AQP1 inhibition will require further studies, based on additional pharmacological evidence using a second, structurally distinct AQP1 inhibitor or genetic approaches, such as AQP1 knockdown or knockout. In the case of this XTCC model or other 3D models, however, this meets certain problems: if using knockout cells, the AQP1 knockout might well interfere with other cellular processes hampering the establishment of tumor xenografts required for setting up the XTCC model. An approach aiming at an siRNA-mediated knockdown would have to rely the direct transfection of the tissue in the XTCC setting. For this strategy, normal transfection reagents cannot be used. For this reason, polymeric nanoparticles for direct siRNA delivery and transfection of tissue slices are currently being developed in our group. Preliminary data indicate that certain polyethylenimine (PEI)-based nanoparticles with defined chemical modifications may lead to sufficient transfection efficacy (unpublished). The major limiting step is the penetration of the nanoparticles into the tissue, for reaching cells that are not on the surface of the tissue slice. *Nota bene*, these deeper cell layers are the most important ones when assessing tumor cell invasion. Once established, it will provide a valuable extension of the model described in this paper.

Expanding on our first evaluation of the XTCC models as a powerful drug screening system [25], our investigation in this study further validates the models as a reliable tool for assessing novel pharmacological agents and distinguishing between effects on bulk tumor mass versus tumor cell invasion. In contrast to the previously described tissue slice tandem model relying on spheroids, the use of xenograft tumors also includes cell types other than tumor cells, thus mimicking more closely the *in vivo* situation and the cell type heterogeneity within a tumor. Notably, in the case of U87-MG cells this yielded substantially higher invasion capacities of the tumor cells as compared to spheroids, as shown previously [24]. Thus, this approach also differs from injection of GBM cells directly into the brain parenchyma.

All the compounds tested here reduced cell invasion to a greater extent than the bulk tumor mass. The results of the XTCC models thus support the notion that these compounds predominantly affect cell invasion rather than bulk tumor growth in GBM cells, which could address a pivotal problem of spread and recurrence in glioblastoma.

Despite the solid tissues (i.e., the tumor xenograft and the normal brain tissue) restricting compound penetration over the relatively short 8-day time period of the experiments, comparatively low concentrations – in some cases only 10 µM – were sufficient for inhibiting or even abolishing tumor cell invasion. The fact that simple cell assays, such as 2D adherent cell culture or 3D spheroids behave differently than more complex tissue-like cell assemblies [42] prompted us to develop the XTCC models [24]. The direct comparison of the XTCCs with scratch assays or spheroid outgrowth assays revealed that the simpler *in vitro* assays, may sometimes rather underestimate inhibitory effects. This suggests that an intact tissue architecture of the tumor as well as of the normal brain tissue, including the other, non-tumor cell types, is required for adequately monitoring invasion processes and inhibitory compound effects. However, one limitation of the models still lies in the fact that, due to the necessity of using immunodeficient mice, the contribution of immune cells cannot be readily monitored. To circumvent this problem, humanized mice or murine cell lines in an immunocompetent mouse model could be employed. Additionally, co-culture models using brain or cancer organoids could be beneficial to analyse effects of immune cells and pre-defined cell interactions, since specific immune cells can be added to the organoid culture allowing for a more refined control of cell composition in contrast to xenograft-based models [43–45]. However, tumor xenograft models display a more realistic tumor environment, including stromal interactions, hypoxia, and vascularization, making it more suitable for drug treatment studies and monitoring invasion behaviour over time [25, 46]. Thus, xenograft tumors were used for examining treatment responses of compounds and translational validation of *in vitro* studies with the same cell lines.

Still, while the XTCCs are able to model certain aspects related to the *in vivo* situation, they can never fully recapitulate the complexity of the *in vivo* setting. This model is at the interface between pure 2D cell culture and *in vivo* experiments, allowing for monitoring certain aspects of the *in vivo* situation and thus for experiments that will identify lead candidates for drug development, prior to their further analysis *in vivo*. While this model thus contributes to the 3R principle, it will not be able to substitute eventual *in vivo* studies.

Our results also suggest the relevance of combination therapies in the context of targeted inhibitors as well as established cytostatics (TMZ). This may be even more important when considering the brain as a relatively deep pharmacological compartment, with major delivery and penetration barriers including the blood-brain barrier (BBB). As a preliminary BBB permeability assessment of the compounds, SwissADME profiling was used. Indeed, all RAD compounds are likely to pass the BBB, while AqB011 has drug-like parameters but was not predicted to offer a high BBB permeability. Thus, the RAD compounds seem to be more promising compared to AqB011 for further development and future *in vivo* studies, which will be necessary to determine BBB permeability conclusively. However, considering that even compounds, which are able to penetrate the BBB may not necessarily reach therapeutic levels at their site of action, the additive or synergistic effects of drug combinations may be particularly relevant. In this context, the availability of compounds which may show lower side effects by not acting through cytotoxic mechanisms but acting as cytostatics, may be particularly attractive for combination therapies. Thus, further work will reveal the relevance of different combinations in *ex vivo* and *in vivo* models.

## Conclusion

Research here reveals the potent inhibition of GBM tumor cell invasion into normal adjacent brain tissue by a set of novel indole compounds and the aryl sulfonamide AqB011. The XTCC models allowed monitoring *ex vivo,* that uncovered how effectively the inhibitors were able to restrain or abolish the ability of GBM cells to invade through the complex environment of viable brain tissue. The full roster of targets which are regulated by the anti-invasive compounds described here remains to be defined, but AQP1 is emerging as one of the candidates of interest. The indole analog RAD118, which has been shown to inhibit AQP1 water channels [21], and AqB011, which blocks AQP1 ion channels [26], were the most effective compounds in decreasing or abolishing invasiveness without affecting cell proliferation or causing apoptosis significantly. These findings may pave the way for urgently needed novel treatment options for GBM patients.

## Supplementary Information

Supplementary Material 1: Chemical structures of the indole parent compound RAD114 and the semisynthetic analogs RAD118, RAD119, RAD120 (upper panel), and the bumetanide derivative AqB011 (lower panel)

Supplementary Material 2: Dose-response curves of viability for treatments with DMSO (0.0001–1%) and indole analogs (0.01–100 μM) measured with WST-8 after 72 h of incubation using (**A**) U87-MG and (**B**) G55T2 cells. Results are from at least two biological replicates with 6 to 9 technical replicates

Supplementary Material 3: Expression of AQP1 relative to housekeepers (*hActin*, *RPLP0*) in U87-MG and G55T2 tumor xenografts (left) and cell lines (right). Results are from one biological experiment with two technical replicates

Supplementary Material 4: Treatment effects of the indole analogs and AqB011 on cell proliferation and apoptosis in XTCCs. (**A, B**) Compiled data in swarm plots (mean ± SD) show the quantification of the total proliferative cell counts in Ki-67-stained FFPE sections from G55T2 and U87-MG XTCCs treated with 1% DMSO or 100 µM test compounds. (**C, D**) Compiled data in swarm plots (mean ± SD) show the quantification of the total apoptotic cell counts in active Caspase-3-stained FFPE sections from G55T2 and U87-MG XTCCs treated with 1% DMSO or 100 µM test compounds. Data were compiled from at least two biological replicates. The numbers of technical replicates are shown above the x-axis. Statistically significant differences between treatments and vehicle control were determined with one-way ANOVA using Dunnett’s multiple comparisons test, ns = not significant. Asterisks indicate statistically significant differences (*, p < 0.05; **, p < 0.01; ***, p < 0.001 and ****, p < 0.0001)

Supplementary Material 5: Immunohistochemical staining of U87-MG XTCCs for phosphorylated p42/44, indicating activation of the ERK pathway

Supplementary Material 6: Immunohistochemical staining of G55T2 XTCCs for phosphorylated p42/44, indicating activation of the ERK pathway

## Data Availability

The data that support the findings of this study are available from the corresponding authors upon reasonable request.

## Abbreviations

ALI: Air-liquid interface
AQP1: Aquaporin-1
BBB: Blood-brain barrier
BSA: Bovine serum albumin
DMSO: Dimethyl sulfoxide
FBS: Fetal Bovine Serum
FFPE: Formalin-fixed Paraffin-embedded
GBM: Glioblastoma/glioblastoma multiforme
PI: Propidium Iodide
RAD: Rohan A Davis
TC: Tissue culture
TMZ: Temozolomide
XTCC: Xenograft tissue slice tandem co-culture
